# The Genetic Loci Associated with Fiber Development in Upland Cotton (*Gossypium hirsutum* L.) Were Mapped by the BSA-Seq Technique

**DOI:** 10.3390/plants14172804

**Published:** 2025-09-07

**Authors:** Yanlong Yang, Fenglei Sun, Xin Wei, Zhengzheng Wang, Jun Ma, Dawei Zhang, Chunping Li, Chengxia Lai, Guoyong Fu, Youzhong Li

**Affiliations:** 1 Xinjiang Key Laboratory of Cotton Genetic Improvement and Intelligent Production, National Cotton Engineering Technology Research Center, Cotton Research Institute of Xinjiang Uyghur Autonomous Region Academy of Agricultural Sciences, Urumqi 830091, China; yangyl0629@163.com (Y.Y.);; 2 Cotton Research Institute, Xinjiang Academy of Agricultural and Reclamation Science, Key Laboratory of Cotton Biology and Genetic Breeding in the Northwest Inland Cotton Production Region, Ministry of Agriculture and Rural Affairs, Shihezi 832000, China

**Keywords:** chromosome segment substitution lines, fiber development, BSA-Seq, quantitative trait loci, candidate gene mapping

## Abstract

Cotton fiber quality improvement remains a fundamental challenge in breeding programs due to the complex genetic architecture underlying fiber development. The narrow genetic base of upland cotton (*Gossypium hirsutum* L.) and the quantitative nature of fiber quality traits necessitate innovative approaches for identifying and incorporating superior alleles from related species. We developed a BC_6_F_2_ population by introgressing chromosome segments from the sea island cotton variety Xinhai 36 (*G. barbadense*) into the upland cotton variety Xinluzhong 60 (*G. hirsutum*). Based on fiber strength phenotyping, we constructed two DNA bulks representing extreme phenotypes (20 superior and 12 inferior individuals) for bulked segregant analysis sequencing (BSA-Seq). High-throughput sequencing generated 225.13 Gb of raw data with average depths of 20× for parents and 30× for bulks. SNP calling and annotation were performed using GATK and ANNOVAR against the upland cotton reference genome (TM-1). BSA-Seq analysis identified 13 QTLs primarily clustered within a 1.6 Mb region (20.6–22.2 Mb) on chromosome A10. Within this region, we detected nonsynonymous mutation genes involving a total of six genes. GO and KEGG enrichment analyses revealed significant enrichment for carbohydrate metabolic processes, protein modification, and secondary metabolite biosynthesis pathways. Integration with transcriptome data prioritized *GH_A10G1043*, encoding a β-amylase family protein, as the key candidate gene. Functional validation through overexpression and RNAi knockdown in *Arabidopsis thaliana* demonstrated that *GH_A10G1043* significantly regulates starch content and β-amylase activity, though without visible morphological alterations. This study successfully identified potential genomic regions and candidate genes associated with cotton fiber strength using chromosome segment substitution lines combined with BSA-Seq. The key candidate gene *GH_A10G1043* provides a valuable target for marker-assisted selection in cotton breeding programs. Our findings establish a foundation for understanding the molecular mechanisms of fiber quality formation and offer genetic resources for developing superior cotton varieties with enhanced fiber strength.

## 1. Introduction

Cotton, as a globally important economic and natural fiber crop, serves as the primary raw material for the textile industry, providing approximately 35% of the world’s total fiber consumption [1]. Although China ranks among the world’s major cotton-producing countries, the production of high-quality cotton remains critically insufficient. Therefore, improving fiber quality continues to be a paramount objective in Chinese cotton breeding programs. Upland cotton (*Gossypium hirsutum* L.) exhibits a narrow genetic base, and both yield and quality traits are quantitative traits controlled by multiple genes, making simultaneous improvement in fiber yield and quality particularly challenging [2]. Traditional hybrid breeding and systematic selection approaches are not only time-consuming and labor-intensive but also face significant difficulties in effectively advancing the cultivation of high-quality cotton varieties [3].

The advent of resequencing technology and the availability of cotton reference genomes [4,5] have enabled rapid evaluation and identification of the genetic basis underlying cotton agronomic traits. Bulked Segregant Analysis (BSA) represents a forward genetic approach and practical gene/marker mapping technique for identifying genomic regions harboring loci that influence target traits. This method involves constructing bulked DNA pools based on extreme phenotypes within genetically segregating populations, followed by screening molecular markers associated with the phenotype through differences in mutation frequency between pools, thereby providing precise localization signals [6]. This approach is recognized as a rapid and accurate method for localizing quantitative trait loci (QTLs) or mining candidate genes for target traits [7], with various computational methods proposed for functional gene localization [8]. First proposed by Michelmore et al. [9] for application in a segregating lettuce population, this simple, efficient, rapid, and cost-effective trait mapping method offers high genomic resolution for selective genotyping of target traits [10]. Currently, BSA-Seq applications have expanded from model to non-model species, with the technology experiencing rapid development and widespread adoption in gene mining research across various crops and plant species [11,12,13,14,15,16]. In cotton, BSA-Seq has successfully mapped genes controlling oil content [17], virescent mutation [18], fruiting branch type [19,20], first fruiting branch node [21], and early maturity [22].

Conventionally, QTL mapping utilizes segregating populations such as F_2_, BC_1_, DH, or RIL [23,24]. However, the complex genetic backgrounds of these populations, coupled with the inherent complexity of quantitative traits and susceptibility to interference from non-target QTLs, severely constrain fine-scale analysis of target traits. Chromosome segment substitution lines (CSSLs) represent ideal materials for QTL fine mapping and complex agronomic trait analysis, effectively overcoming the limitations of interspecific hybridization segregating populations in gene introgression. They effectively eliminate genetic background noise and enable the prediction of QTLs that may be masked in primary populations, such as F_2_ or RIL [25]. Currently, chromosome segment substitution lines have been extensively utilized for in-depth research across different crops, including rice [26], soybean [27], rapeseed [23], and cotton [28]. Upland cotton (*Gossypium hirsutum* L.) accounts for over 90% of global annual cotton production due to its high yield potential and broad adaptability, though its fiber quality remains moderate. Sea island cotton (*Gossypium barbadense* L.) has garnered considerable attention for its superior fiber quality and disease resistance [4,5,29]. The land–sea introgression lines, representing high-generation backcross populations, contain predominantly upland cotton genetic background with limited segments from sea island cotton. This simplified genetic background yields more accurate QTL mapping results, making them optimal materials for the fine mapping of fiber quality trait QTLs in upland cotton while simultaneously broadening the narrow genetic base of upland cotton cultivars. Researchers have identified numerous QTLs associated with cotton yield, fiber quality, and Verticillium wilt resistance using CSSL populations. In 2005, Stelly et al. [30] pioneered the construction of land–sea introgression line populations using conventional breeding techniques, including hybridization, backcrossing, and selfing, obtaining 17 germplasm lines containing sea island cotton 3–79 chromosome segments within the upland cotton TM-1 genetic background. Based on the land–sea introgression line population constructed from upland cotton TM-1 and sea island cotton 3–79, Luan et al. [31] developed secondary segregating populations using recurrent parent TM-1 with CS-B14Sh and CS-B22Sh, identifying 24 QTLs related to yield or fiber quality traits in F_2_ and F_2:3_ populations. Shi et al. [32,33] constructed a land–sea introgression line population comprising 408 lines using upland cotton CCRI 36 and sea island cotton Hai 1 as parents. Through integration of high-density genetic linkage maps and multi-year, multi-environment phenotypic data, they identified 227 QTLs associated with yield, fiber quality, and Verticillium wilt resistance. Therefore, utilizing chromosome segment substitution lines to introduce superior fiber quality genes from sea island cotton into upland cotton and exploring important QTLs related to fiber quality traits provides not only excellent genetic resources for upland cotton breeding but also novel approaches for comprehensive aggregation of superior traits from both species, establishing a material foundation for developing new cotton varieties with both high yield and superior quality.

There are several recent reports on the application of BSA-Seq for localizing QTLs or genes for cotton-related traits using land–sea introgression lines. Wang et al. [24] constructed 169 chromosome segment introgression lines using upland cotton genetic standard line TM-1 and sea island cotton Hai7124, detecting 22 molecular markers associated with fiber quality in the BC_5_S_1_ population and thereby establishing a foundation for fine mapping of fiber quality QTLs using cotton chromosome segment introgression lines. Cao et al. [34] utilized sea island cotton chromosome segment introgression lines with Xinluzao 41 background as materials, successfully localizing QTLs for upper half mean length, fiber strength, and micronaire value within 1cM intervals. While significant progress has been achieved in QTL mapping for fiber quality, direct applications to cotton quality improvement remain relatively limited. The primary constraint is the scarcity of stable QTL segments and major novel genes [35]. Therefore, further exploration and validation of fiber quality QTLs remain urgently needed. In this study, to explore key candidate genes related to cotton fiber quality, we constructed Xinhai 36 chromosome segment introgression lines with a Xinluzhong 60 background and conducted comprehensive testing and statistical analysis of key quality traits in the BC_6_F_2_ population. Based on these analyses, we selected individuals exhibiting higher and lower fiber strength to construct extreme bulks, followed by BSA-Seq resequencing of the parents and two extreme bulks. Through comparison of allele frequencies, we localized candidate regions associated with upland cotton fiber strength and performed bioinformatics analysis of candidate genes within these regions. Our objective was to screen genomic loci and candidate genes related to fiber quality, thereby establishing a foundation for molecular marker-assisted breeding of high-quality cotton varieties.

## 2. Results

### 2.1. Statistical Analysis of Fiber Quality Traits

**Figure 1:**
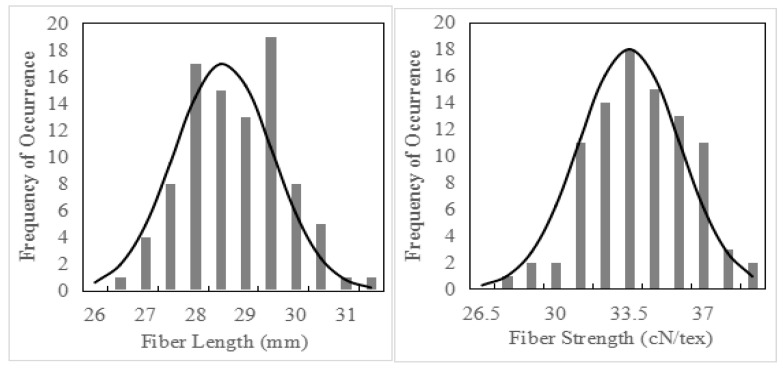
Fiber quality frequency distribution of the BC_6_F_2_ population.

The phenotypic trend of fiber quality in the two ecological regions is basically the same, so the average value of fiber quality data in the two ecological regions is selected for analysis. The initial analysis revealed significant differences in fiber quality between the two parents, especially in fiber length and fiber strength (Table 1). Following harvest, we employed IBM SPSS Statistics 19.0 to generate fiber quality frequency distribution plots for the population and conducted normality tests. The results demonstrated that both skewness and kurtosis values were less than 1, indicating that fiber length and fiber strength in the population conformed to normal distributions. Based on these data, we clarified that the fiber quality of this population was a quantitative trait (Table 2, Figure 1). A total of 184 families in the BC_6_F_2_ population were tested. Subsequently, we sorted the fiber quality data from low to high in Excel. According to different fiber length and fiber strength indexes, we selected 20 families with fiber length > 30.5 mm and fiber strength > 31 cN/tex to construct extreme pool 1 (HC1) and selected 12 families with fiber length < 29.5 mm and fiber strength < 30 cN/tex to construct extreme pool 2 (HC2).

### 2.2. BSA-Seq Quality Assessment

Using the Illumina NovaSeq sequencing platform with a PE150 sequencing strategy, we obtained a total of 225,131,098,200 bp of raw data by sequencing the two parents and two extreme bulks. After implementing quality control measures, including adapter removal and low-quality read filtering, we obtained 1,700,873,988 clean bases. Quality assessment revealed that the GC content of these reads ranged from 35.35% to 35.66%, with high sequencing data quality (Q20 ≥ 96.39%, Q30 ≥ 90.81%), suitable for subsequent analyses (Supplementary Table S1).

### 2.3. Mapping Analysis, SNP Detection, and Annotation

After mapping the quality-controlled data to the TM-1 reference genome from the integrated database (https://www.cottongen.org/species/Gossypium_hirsutum/ZJU-AD1_v2.1) (accessed on 1 August 2021), we obtained a total of 1,618,463,526 mapped reads. The results demonstrated sample mapping rates of 99.50–99.66%, with average sequencing depths of 20× for the parents and 30× for the bulked pools, indicating uniform random coverage of the reference genome with high mapping rates conducive to subsequent SNP screening and annotation (Table 3).

### 2.4. Candidate Region Localization and Gene Screening

**Figure 2:**
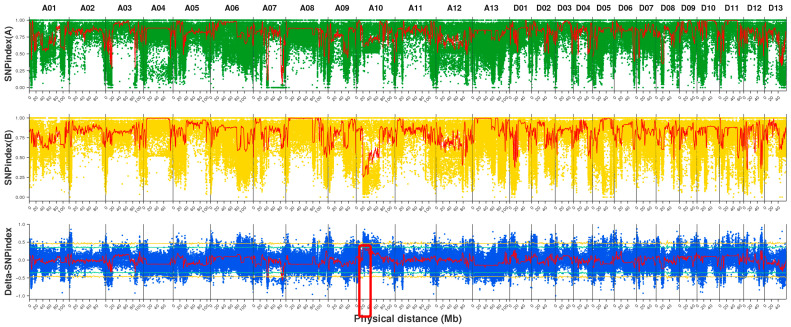
Distribution of SNP-index association values across chromosomes. The abscissa is the name and length of each chromosome, and the ordinate represents the SNP-index value. The point is the SNP-index, the red line is the mean value of the SNP-index under the window, the orange line is the 99% confidence line, and the green line is the 95% confidence line. SNPindexA: Distribution of SNP-index values for HC1 on chromosomes. SNPindexB: Distribution of SNP-index values for HC2 on chromosomes. Delta-SNPindex: Distribution of Δ(SNP-index) values on chromosomes.

To obtain reliable SNPs, we employed the UnifiedGenotyper model in GATK software (4.4.0.0) [36] for SNP detection. Following ANNOVAR annotation [37], we obtained 17,291,395 SNPs (Supplementary Table S2). Among these, 255,107 SNPs were located in exonic regions, including 159,457 nonsynonymous mutations, 3920 stop gain mutations, and 963 stop loss mutations. To visualize the SNP-index distribution across chromosomes, we generated chromosome-wide SNP-index distribution plots (Figure 2). We subsequently calculated the SNP-index difference between the two progeny pools, obtaining Δ(SNP-index) = SNP-index (extreme trait A) − SNP-index (extreme trait B). Following 10,000 permutation tests, we selected windows with confidence levels exceeding 99% as the screening threshold to determine candidate intervals. The genome-wide distribution results of the two offspring pools and Δ(SNP-index) are presented in Figure 2.

Ultimately, we identified a total of 13 positive regulatory loci, predominantly distributed within the 20,600,000 bp to 22,200,000 bp region of chromosome A10 (Figure 2). Further analysis of nonsynonymous mutation sites located in exons within the candidate region revealed a total of seven sites involving six genes (Supplementary Table S3).

### 2.5. GO Classification and Enrichment Analysis

**Figure 3:**
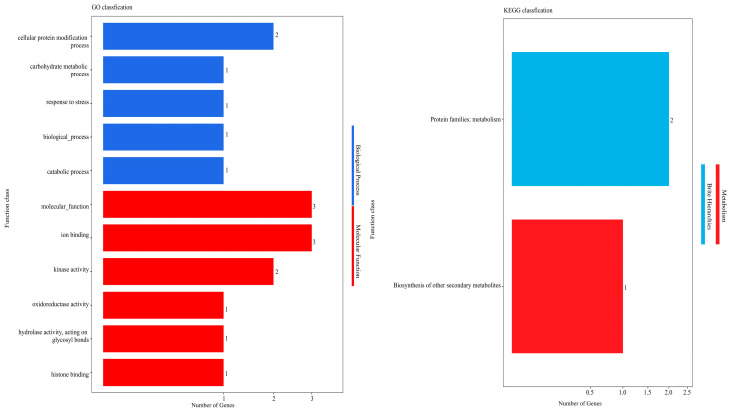
GO enrichment and KEGG pathway annotations of candidate genes.

GO enrichment analysis was conducted on these genes, with classification according to biological process (BP), molecular function (MF), and cellular component (CC). GO functional annotation analysis of the identified six genes revealed annotation into two functional groups (Figure 3), encompassing five biological processes and six molecular functions. Within biological processes, these genes were primarily involved in the cellular protein modification process, carbohydrate metabolic process, and response to stress. For molecular functions, these genes were primarily associated with molecular function, ion binding, and kinase activity. These findings suggest that fiber development processes may be related to cellular protein modification, carbohydrate metabolism, and various biological and catabolic processes, with molecular function, ion binding, and kinase activity potentially playing crucial roles in fiber development.

To identify metabolic pathways enriched among the six genes, we performed pathway enrichment analysis using the KEGG pathway database. The results showed that three of the six genes were primarily enriched in protein families: metabolism and biosynthesis of other secondary metabolites pathways (Figure 3). This indicates that metabolism and biosynthesis of secondary metabolites play significant roles in fiber development.

The KEGG enrichment analysis results were largely consistent with the GO enrichment findings, further supporting the important roles of biosynthesis and metabolic pathways in cotton fiber development.

### 2.6. Functional Analysis of Candidate Genes GH_A10G1043 in Arabidopsis

**Figure 4:**
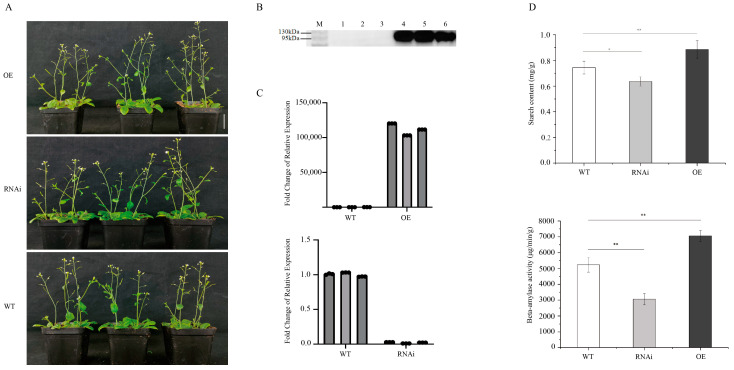
Functional analysis of candidate gene *GH_A10G1043* in *Arabidopsis*. (**A**) Phenotypes of wild-type (WT), overexpression (OE), and knockdown lines (RNAi). (**B**) Western blot analysis of overexpression plants. From left to right: Marker, 1, 2, 3 channels represent pCAMBIA-eGFP empty vector, 4, 5, 6 channels represent pCAMBIA-GHA10G1043-eGFP three overexpression transgenic lines. (**C**) Quantitative RT-PCR (RT-qPCR) validation results. (**D**) Starch content and β-amylase activity in wild-type, overexpression, and knockdown plants. Asterisks indicate significant differences at various thresholds (* *p* < 0.05, ** *p* < 0.01). Error bars represent the mean ± SE of three biological replicates.

In our previous study, “Genome-Wide Identification and Preliminary Functional Analysis of BAM (β-Amylase) Gene Family in Upland Cotton” [38], we performed tissue-specific analysis of all GhBAM family genes combined with transcriptome analysis. We identified two specifically expressed genes, including *GH_A10G1043* and *GH_D10G1788*. Further qRT-PCR verification suggested that *GH_A10G1043* may be involved in fiber quality formation in upland cotton. In the present study, this gene was also identified as a candidate gene related to cotton fiber strength. Due to the long transgenic cycle of cotton, we selected the model plant Arabidopsis thaliana for preliminary functional verification to determine whether the candidate genes need to be further transduced into upland cotton. In order to preliminarily verify the function of the gene, we overexpressed the candidate gene in *Arabidopsis thaliana* and performed RNAi-mediated knockdown of the homologous gene. Phenotypic analysis revealed no visible morphological changes in overexpression or knockdown plants compared to wild-type plants (Figure 4A). Western blot analysis of overexpression lines showed clear bands of the expected size for the target protein (Figure 4B). qRT-PCR analysis of candidate gene expression in wild-type, overexpression, and knockdown plants demonstrated significantly higher expression in overexpression lines compared to wild-type plants, while knockdown lines showed significantly reduced expression relative to wild-type plants (Figure 4C). Furthermore, we measured the starch content and β-amylase activity in wild-type, overexpression, and knockdown *Arabidopsis* plants. Both the starch content and β-amylase activity were significantly lower in knockdown lines compared to wild-type lines, while overexpression lines showed higher levels than wild-type lines. Additionally, the starch content and β-amylase activity were significantly higher in overexpression lines compared to knockdown lines (Figure 4D).

## 3. Discussion

### 3.1. Parent Selection, Population Construction, and Fiber Quality Trait Analysis

Chromosome segment introgression lines (CSILs) represent ideal materials for detecting quantitative trait loci (QTLs). Developed through hybridization, advanced backcrossing, selfing, and marker-assisted selection, these lines contain only one or a few chromosome segments from the donor parent while maintaining a genetic background highly similar to the recurrent parent, thereby eliminating interference from genetic background complexity. Stelly et al. [30] developed the first set of cotton CSILs using the standard upland cotton line TM-1 as the recurrent parent. While the TM-1 genetic background facilitates QTL mapping, its practical application in cotton breeding remains limited. Compared to cotton chromosome segment introgression lines developed by previous researchers [24], this study selected Xinjiang’s recently developed high-quality, high-yield, and disease-resistant sea island cotton variety Xinhai 36 as the donor parent, and Xinjiang’s self-bred, nationally approved upland cotton variety Xinluzhong 60, with superior quality, as the recurrent parent. This approach not only ensures significant fiber quality differences among segregating progeny to meet QTL mapping requirements but also creates germplasm resources with superior comprehensive traits and excellent fiber quality. Additionally, it provides valuable insights into domestication patterns of sea island and upland cotton, as well as strategies for improving upland cotton quality and increasing sea island cotton yield.

Upland cotton (*Gossypium hirsutum*) and sea island cotton (*Gossypium barbadense*) share a common origin but have undergone independent evolution, resulting in significant genomic differentiation and distinct phenotypic differences in plant morphology, yield, and fiber quality [5]. In this study, the BC_6_F_2_ population derived from upland × sea island cotton hybridization exhibited extensive variation in fiber quality traits, with phenotypic values following normal distributions. Chromosome segment substitution lines (CSSLs) represent permanent segregating populations where variation loci are predominantly homozygous, leading to relatively stable genetic composition and high heritability [39]. Such populations with well-defined genetic backgrounds are particularly suitable for QTL mapping analysis, as linkage analysis results are less susceptible to population structure effects. Direct measurement of genotypes and phenotypes in subsequent generations enables the determination of chromosomal locations and genetic effects of genes controlling fiber quality traits through genotype–phenotype correlations. The utility of CSSL populations in improving QTL analysis accuracy has been demonstrated across numerous crop species.

### 3.2. Analysis of BSA-Seq Localization Results

Bulked segregant analysis sequencing (BSA-Seq) represents an advanced technique that targets specific traits by selecting two parents and their progeny exhibiting extreme phenotypic differences to construct DNA pools for whole-genome resequencing. Differential DNA fragments detected between pools represent candidate regions [40]. Compared to traditional sequencing methods, this technology directly utilizes polymorphic SNPs between parents for gene mapping, offering advantages including simplicity, efficiency, rapid and accurate gene localization, and elimination of genetic linkage map construction requirements [12,41]. Consequently, BSA-Seq has been increasingly applied for mapping genes related to agronomic traits in crops. Additionally, BSA-Seq results provide information on mutation sites within candidate regions, facilitating molecular marker design during fine mapping. For instance, Takagi et al. [42] employed BSA-Seq to identify the candidate gene OsRR22 controlling salt tolerance in rice; Lu et al. [43] mapped 84 genes controlling early flowering in cucumber and identified CsFT as a candidate gene; Illa-Berenguer et al. [44] localized 66 genes controlling fruit weight in tomato; Guo et al. [45] rapidly mapped 29 genes associated with cucumber mosaic virus resistance in pepper and identified two candidate genes; Ma et al. [46] identified candidate genes controlling low stigma exsertion mutants in rice; Zhao et al. [47] identified 20 candidate genes for fertility restoration of cytoplasmic male sterility in cotton; and Zhang et al. [48] mapped 8 candidate genes controlling the determinate inflorescence trait in rapeseed.

By utilizing the BC_6_F_2_ introgression line population combined with BSA-Seq analysis, QTLs and candidate genes can be rapidly identified and screened. Upland cotton (*Gossypium hirsutum*) represents one of the most widely cultivated cotton varieties globally, renowned for its high yield and strong adaptability. With advances in high-throughput sequencing technologies, BSA-Seq analysis has been successfully applied for the rapid mining and screening of candidate genes across various crop species.

In this study, we constructed two extreme bulks using upland cotton (*Gossypium hirsutum*) and sea island cotton (*Gossypium barbadense*) materials exhibiting significant differences in fiber strength, aiming to explore key candidate genes controlling cotton fiber strength. Through BSA-Seq, we identified one candidate region on chromosome 10 and involved six candidate genes. Comparison with the literature and analysis of nucleotide sequences and positions revealed that the candidate region and gene loci identified in this study differ from those previously reported. This region, located between 20,600,000 bp and 22,200,000 bp on chromosome 10, covers a relatively large interval containing multiple candidate genes. However, further development of molecular markers or application of bioinformatics methods is required for QTL fine mapping or candidate gene mining. Subsequently, by integrating BSA-Seq results with RNA-seq data, we further narrowed the candidates to one key gene controlling fiber strength for detailed analysis. Fiber strength represents a quantitative trait controlled by multiple genes with a complex genetic basis, often regulated by numerous physiological and molecular factors. Therefore, we believe that continued research on these candidate regions and genes related to fiber strength may play crucial roles in future quality breeding programs. A limitation of this study is the relatively large candidate interval identified. Future work will involve expanding the population size to further narrow the candidate interval. Identification and functional validation of candidate genes within the mapped interval will be analyzed and verified in subsequent studies.

Comparison with QTLs localized by Si et al. [28] and Wang et al. [49] revealed that while QTLs related to fiber quality were mapped to chromosome A10, their physical locations differed. Si et al. [28] mapped qFS-A10-1 (NAU5323, 97.86 Mb) through genetic map construction. Wang et al. [49] mapped qELO-10-1 (JESPR6-BNL1161, 67.02–75.68 Mb) through genetic mapping. Shen et al. [50] constructed a yellow-brown cotton chromosome segment introgression line population, containing 71 families with an upland cotton background, and mapped qFE-A10 (88.40 Mb) through SLAF-seq sequencing combined with genetic mapping. These findings indicate that the A10 chromosome interval makes substantial contributions to fiber development.

### 3.3. GH_A10G1043 as a Candidate Gene Related to Cotton Fiber Strength

In our previous study, “Genome-Wide Identification and Preliminary Functional Analysis of BAM (β-Amylase) Gene Family in Upland Cotton” [38], we performed tissue-specific analysis of all GhBAM family genes in upland cotton combined with transcriptome analysis. We identified two specifically expressed genes, including GH_A10G1043, which was further validated by qRT-PCR. These analyses suggested that GH_A10G1043 may be involved in fiber quality formation in upland cotton.

In the present study, *GH_A10G1043* was also identified as a candidate gene related to cotton fiber strength, with a genomic DNA length of 2512 bp and CDS sequence length of 1611 bp. GH_A10G1043 belongs to the β-amylase (*BAM*) family, members of the glycosyl hydrolase 14 family characterized by a conserved glycosyl hydrolase 14 domain. This family is widely distributed across various plants and some microorganisms and is encoded by a multi-gene family. Its homologous protein in *Arabidopsis* was annotated as inactive *BAM9*. *BAM9* represents one member of the *BAM* family, which comprises important enzymes catalyzing the conversion of plant starch into maltose. As the only plant enzyme capable of producing β-maltose, it plays crucial roles in regulating plant growth and development. β-amylase (*BAM*) functions as an exoamylase, catalyzing the hydrolysis of α-1,4-linked oligosaccharides and polyglucans. It is primarily responsible for the hydrolysis of storage starch and the degradation of transitory starch, yielding β-limit dextrin and β-maltose [51]. However, this family also includes proteins with weak catalytic activity, additional domains, or lacking colocalization with starch substrates. Recent *Arabidopsis* studies have demonstrated that *BAM9*, despite being a catalytically inactive chloroplast protein, possesses unique regulatory functions in starch metabolism. It facilitates carbohydrate utilization by promoting starch degradation [52] or interacts with other amylases to enhance their activity, thereby promoting starch degradation and providing additional energy sources for physiological metabolic processes under stress conditions [53]. Future work will focus on in-depth functional analysis and molecular marker development to verify mutation sites in other populations, with the goal of distinguishing materials with superior versus inferior fiber strength through genotyping, thereby providing potential application value in breeding high-quality cotton varieties.

## 4. Materials and Methods

### 4.1. Plant Materials

The experimental cotton variety of Xinluzhong 60 (P60) is a nationally approved upland cotton variety with superior quality and high specific strength. Xinhai 36 (P36) is a high-quality, disease-resistant sea island cotton variety. Both varieties were developed by the Agricultural Science Research Institute of the First Division of Xinjiang Production and Construction Corps and Xinjiang Tarim River Seed Industry Co., Ltd. In this study, P60 and P36 were acquired from a variety breeding institution and approved by the Professional Committee of the Main Crop Variety Approval Committee of Xinjiang Uygur Autonomous Region. The phenotypic values of fiber quality refer to the results released by the Xinjiang Variety Approval Committee. Both island cotton and upland cotton are allotetraploid cotton cultivars. Before this experiment, we developed a set of Xinhai 36 chromosome segment introgression lines with a Xinluzhong 60 background. The development process was as follows: In summer 2014, F_1_ hybrids were produced by crossing Xinluzhong 60 × Xinhai 36 at the Kuqa experimental base of Xinjiang Academy of Agricultural Sciences. In the winter of the same year, BC_1_ was obtained by backcrossing F_1_ with Xinluzhong 60 in Sanya, Hainan. In summer 2015, BC_1_ plants were grown in Kuqa City, Xinjiang, and BC_2_ was obtained through backcrossing with Xinluzhong 60. Subsequently, backcrossing with Xinluzhong 60 was performed annually in Kuqa City, Xinjiang, until BC_6_F_1_. In 2020, the BC_6_F_1_ introgression line population was planted in Kuqa, and the BC_6_F_2_ population was produced through selfing.

### 4.2. Field Planting and Trait Investigation

In 2021, the 184 BC_6_F_2_ introgression lines were planted in two ecological areas: Shihezi City and Kuqa City, Xinjiang. Shihezi City and Kuqa City in Xinjiang represent two similar ecological zones in southern and northern Xinjiang. Planting in the two ecological zones was conducted to verify the accuracy of the fiber quality phenotype. Each family was planted in two rows with 5.00 m row length and 0.12 m plant spacing. The planting pattern adopted was (66 + 10 + 66) cm. At the seedling stage, parents and families were numbered, and fresh young leaves were collected, immediately frozen in liquid nitrogen, and stored at −80 °C for DNA extraction. Standard field management practices were implemented throughout the growth period [54]. After harvest, each family was ginned, and at least 20 g of lint cotton was collected. Fiber quality testing was conducted by the Cotton Quality Supervision and Inspection Test Center of the Ministry of Agriculture and Rural Affairs (Urumqi, Xinjiang). The measured parameters included fiber upper half mean length/fiber length (FL) and fiber strength (FS) [54]. We analyzed fiber quality data for each family using SPSS 19.0 software and selected families with higher and lower fiber length and fiber strength using Excel software to construct extreme bulks. Extreme bulk A(HC1) represents the traits of higher fiber length and fiber strength. Extreme bulk B(HC2) represents the traits of lower fiber length and fiber strength.

### 4.3. BSA-Seq

#### 4.3.1. DNA Extraction, Library Construction, and Sequencing

Genomic DNA was extracted using a Plant Genomic DNA Kit (TIANGEN Biotech, Beijing, China), and quality was assessed using a NanoDrop 2000C Spectrophotometer (Thermo Scientific, Waltham, MA, USA) and agarose gel electrophoresis to ensure library construction requirements were met. The minimum concentration of the sample DNA library was 2.5 ng/μL, the total amount of the single library was 0.2 μg, and the volume was greater than 15 μL. Qualified samples were submitted to Nanjing Paisennuo Gene Technology Co., Ltd. (Nanjing, China), for DNA library construction, PCR amplification, and purification. The resulting libraries were sequenced on the high-throughput sequencing platform (Illumina NovaSeq; Illumina, San Diego, CA, USA). Sequencing depth was >20× for parents and >30× for offspring pools.

#### 4.3.2. Reference Genome Alignment, SNP Detection, and Annotation

The quality of the original sequencing data was evaluated, and clean reads were obtained by filtering the original data using the sliding window method using fastp software (v0.20.0). High-quality filtered data were aligned to the reference genome (https://www.cottongen.org/species/Gossypium_hirsutum/ZJU-AD1_v2.1 (accessed on 1 August 2021)) using the bwa (0.7.12-r1039) [36] mem program with default parameters. Based on the alignment results, the IndelRealigner command in GATK was used to realign all reads near InDels to improve SNP prediction accuracy. GATK software (4.4.0.0) [37] was employed for SNP detection, and ANNOVAR [55] software was used for SNP annotation.

#### 4.3.3. Candidate Region Analysis and Gene Identification

SNP frequency (SNP-index) was calculated for the two extreme bulks (HC1 and HC2), with the parents (P60 and P36) serving as a reference. SNP-index values of 0 indicated the same as the parent, while values of 1 indicated a complete difference from the parent. To minimize the impact of sequencing and alignment errors, we filtered polymorphic loci after calculating the SNP-index, removing loci with SNP-index values less than 0.5 and SNP depth values less than 5× in both pools, as well as loci missing in either pool. The difference in allele frequency between the two extreme bulks was calculated as Δ(SNP-index) = SNP-index (bulk A; HC1) − SNP-index (bulk B; HC2). Finally, 10,000 permutation tests were performed, with a 99% confidence level selected as the threshold for screening candidate intervals and loci.

All genes identified within the associated regions were annotated. First, BLAST software (2.14.0) [56] was used to perform comprehensive annotation through multiple databases, including NR [57], Swiss-Prot, GO [58], KEGG [59], and COG [60], for coding genes within candidate intervals. In principle, all genes within the genomic candidate interval are considered candidate genes [61], though further screening and identification of candidate genes through detailed annotation are required. We performed GO annotation of genes harboring mutations within the candidate region using InterProScan software (5.48-83.0).

### 4.4. Gene Transformation and Identification

#### 4.4.1. Arabidopsis Thaliana Transformation

The full-length coding sequence of *GH_A10G1043* was amplified from cDNA by PCR using the gene-specific primers listed in Supplementary Table S4. Among them, a homologous recombination kit was used to connect the recombinant vector, and the cDNA was connected to the vector by double enzyme digestion. PCR products were cloned into pCAMBIA2300-GFP and pUB-GFP-RNAi-Kan vectors driven by the constitutive Cauliflower mosaic virus 35S promoter. The 35S:GH_A10G1043 vector was introduced into *Agrobacterium tumefaciens* and subsequently transformed into *Arabidopsis* plants (Columbia-0, Col-0) using the floral-dip method [62]. Transgenic plants were selected on solid Murashige and Skoog (MS) medium plates containing 50 μg/mL kanamycin and grown under 16 h (22 °C)/8 h (20 °C) light/dark photoperiod. After surface sterilization, seeds from transformed plants were sown on 1/2 MS medium supplemented with kanamycin (50 μg/mL) for selection. Selected individuals were transplanted to soil. Following seed harvest, selection was repeated through three generations to obtain three different homozygous lines. After selection, total membrane proteins were extracted, and target gene overexpression was confirmed by Western blot using Flag antibody.

#### 4.4.2. Western Blot Analysis of Overexpression Plants

Fresh leaf samples of transgenic Arabidopsis positive seedlings at 14 days of age were collected according to the method of Mahmood et al. [63], frozen in liquid nitrogen, and ground into powder. The powder was transferred to pre-cooled 2 mL centrifuge tubes, and protein extraction buffer was added. The samples were boiled for 5 min and centrifuged at 12,000 rpm for 5 min; then, the supernatant was collected for protein extraction. The protein concentration was detected by a protein quantification kit (BCA method). The kit was an Abbkine protein quantification kit (KTD3001). The specific process is shown in the instructions. The loading amount was calculated according to the protein concentration to ensure that the loading amount of each well was consistent.

Following protein extraction, polyacrylamide gel electrophoresis was performed (the gel composition is shown in Table 4), followed by transfer to a PVDF membrane for blocking, incubation, and washing before ECL chemiluminescence development. The PVDF membrane was placed in the detection buffer to balance for 5 min and then placed on the fresh-keeping membrane. An equal volume of liquid A and liquid B in the appropriate amount of the ECL kit was mixed and added to the surface of the membrane, which was then moved into the gel imaging analyzer for chemical photosensitive mode exposure development. The YEASEN chemiluminescence hypersensitive chromogenic kit (36208ES60) was used.

#### 4.4.3. Quantitative RT-PCR (RT-qPCR) Validation

Total RNA was extracted from *Arabidopsis* leaves for RT-qPCR analysis. Following the manufacturer’s instructions, we used a PrimeScript™ RT kit with gDNA Eraser for cDNA reverse transcription. Primers were designed using Primer 5 software, with actin serving as the internal reference gene (Supplementary Table S5) and the concentration and volume of RNA per tube used in qPCR (Supplementary Table S6). The fluorescence quantitative instrument was an abi7500 fluorescence quantitative PCR instrument, using the Tianlong Gentier 96E/96R automatic medical PCR analysis system. RT-qPCR analysis was performed on all samples using SYBR^®^ Premix Ex Taq™ II (Tli RNaseH Plus) on a fluorescence quantitative PCR instrument (ABI7500, Applied Biosystems, Foster City, CA, USA). Each reaction included at least 3 biological replicates, with 3 technical replicates per biological replicate. The relative expression levels of target genes were calculated using the 2^−∆∆Ct2^ method [64].

#### 4.4.4. Determination of Starch Content and β-Amylase Activity

Leaf samples of 14-day-old Arabidopsis thaliana were collected and processed according to the instructions of the starch content kit. Starch was hydrolyzed to glucose by acid hydrolysis, followed by glucose content determination using anthrone colorimetry, allowing for the calculation of the starch content. The determination wavelength was 620 nm. β-amylase activity was measured according to the β-amylase (β-AL) kit’s instructions. α-amylase activity was inactivated by heating at 70 °C, and β-amylase activity was calculated by comparing the total activity (α + β) measured under non-inactivation conditions.

## 5. Conclusions

Cotton, as a natural textile fiber crop and important cash crop that provides raw materials for the textile industry, holds strategic importance in China’s national economy. Exploring fiber quality genes and quality formation mechanisms in upland cotton represents a crucial approach for promoting healthy and sustainable development of the cotton textile industry. In this study, we used the land–sea introgression line population to initially identify the nonsynonymous mutation candidate region by BSA-Seq technology, involving six genes. Through RNA-Seq and qRT-PCR validation, we initially identified a key candidate gene that may be related to cotton fiber strength. Combined with previous studies, we hypothesize that starch metabolism may play an important role in cotton fiber strength formation. However, further validation is required to confirm these conclusions. Therefore, future studies will validate the candidate genes and our hypothesis through transgenic approaches. The comprehensive information provided here offers a reference for understanding cotton fiber strength formation mechanisms and provides the key candidate gene *GH_A10G1043* related to fiber strength, establishing a foundation for further QTL fine mapping and marker-assisted selection breeding.

## Figures and Tables

**Table 1:** Phenotypic value of fiber quality of parents and extreme pools.

Parents	Fiber Length/mm	Fiber Strength/cN·Tex^−1^	Micronaire Value	Remark
Xinluzhong 60	29.10	33.60	4.80	Gossypium hirsutum
Xinhai 36	38.70	46.70	4.50	Gossypium barbadense
HC1	31.10	34.20	4.57	higher fiber length and fiber strength
HC2	28.40	29.72	4.69	lower fiber length and fiber strength

**Table 2:** Statistical analysis of fiber quality of the BC_6_F_2_ population.

	Mean Value	Standard Deviation	Coefficient of Variation	Skewness	Kurtosis	Maximum Value	Minimum Value	Range
Fiber length (mm)	29.56	1.18	3.98	0.07	−0.38	32.71	26.73	5.98
Fiber strength (cN/tex)	33.69	2.46	7.32	−0.20	−0.19	38.70	26.70	12.00

**Table 3:** Quality statistics of mapping with the reference genome for BSA-Seq.

Sample	Mapped Reads	Total Reads	Mapping Rate (%)	Average Depth (×)	Coverage 1 (%)	Coverage 4 (%)
P36	321,236,430	322,838,918	99.50	16.19	93.44	88.63
P60	324,778,375	325,993,176	99.63	16.75	99.07	97.62
HC1	485,444,823	487,109,780	99.66	25.46	99.57	98.76
HC2	487,003,898	488,691,023	99.65	25.14	99.60	98.75

P36: Xinhai 36; P60: Xinluzhong 60; HC1: extreme bulk A; HC2: extreme bulk B. Mapped reads: the total number of reads on the reference genome was compared. Total reads: the total reads of valid sequencing data. Mapping rate: the number of reads on the reference genome was compared to the number of reads in the valid sequencing data. Average depth: the average sequencing depth, the total number of bases compared to the reference genome divided by genome size. Coverage 1: the proportion of bases whose coverage depth is not less than 1 in the whole genome. Coverage 4: the base coverage depth in the whole genome is no less than the base ratio of 4.

**Table 4:** Preparation of 10% separation gel and 5% concentrated gel (mL).

Reagent	10% Separation Gel	5% Concentrated Gel
H_2_O	4.1	2.8
1.5 mol/L Tris-HCl (PH8.8)	2.5	-
1.5 mol/L Tris-HCl (PH6.8)	-	0.51
30% Acryamide	3.3	0.67
10% SDS	0.1	0.05
10% AP	0.1	0.04
TEMED	0.005	0.003

## Data Availability

The data are contained within this article.

## Supplementary Materials

The following supporting information can be downloaded at https://www.mdpi.com/article/10.3390/plants14172804/s1: Table S1: The quality summary of BSA-Seq data; Table S2: SNP_Annotation_Statistics; Table S3: The basic situation of 6 candidate genes; Table S4: The gene-specific primers; Table S5: Candidate gene RT-qPCR primer design; Table S6: The amount of RNA per one tube used in qPCR.
